# C-C motif-ligand 2 inhibition with emapticap pegol (NOX-E36) in type 2 diabetic patients with albuminuria

**DOI:** 10.1093/ndt/gfv459

**Published:** 2016-04-27

**Authors:** Jan Menne, Dirk Eulberg, Diana Beyer, Matthias Baumann, Frantisek Saudek, Zsuzsanna Valkusz, Andrzej Więcek, Hermann Haller, for the Emapticap Study Group

**Affiliations:** 1 Department of Nephrology and Hypertension, Hannover Medical School, Hannover, Germany; 2 NOXXON Pharma AG, Berlin, Germany; 3 Institute for Clinical and Experimental Medicine, Prague, Czech Republic; 4 Albert Szent-Györgyi Health Center, University of Szeged, Szeged, Hungary; 5 Department of Nephrology, Endocrinology and Metabolic Diseases, Medical University of Silesia, Katowice, Poland

**Keywords:** albuminuria, diabetes mellitus, diabetic nephropathy, inflammation, macrophage

## Abstract

**Background:** Emapticap pegol (NOX-E36) is a Spiegelmer^®^ that specifically binds and inhibits the pro-inflammatory chemokine C-C motif-ligand 2 (CCL2) (also called monocyte-chemotactic protein 1). The objective of this exploratory study was to evaluate the safety and tolerability as well as the renoprotective and anti-diabetic potential of emapticap in type 2 diabetic patients with albuminuria.

**Methods:** A randomized, double-blind, placebo-controlled Phase IIa study was initiated in 75 albuminuric type 2 diabetics. Emapticap at 0.5 mg/kg and placebo were administered subcutaneously twice weekly for 12 weeks to 50 and 25 patients, respectively, followed by a treatment-free phase of 12 weeks.

**Results:** Twice weekly subcutaneous treatment with emapticap over 3 months was generally safe and well tolerated and reduced the urinary albumin/creatinine ratio (ACR) from baseline to Week 12 by 29% (P < 0.05); versus placebo a non-significant ACR reduction of 15% was observed (P = 0.221). The maximum difference, 26% (P = 0.064) between emapticap and placebo, was seen 8 weeks after discontinuation of treatment. At Week 12, the HbA1c changed by −0.31% in the emapticap versus +0.05% in the placebo group (P = 0.146). The maximum difference for HbA1c was observed 4 weeks after the last dose with −0.35% for emapticap versus +0.12% for placebo (P = 0.026). No relevant change in blood pressure or estimated glomerular filtration rate was seen between the treatment groups throughout the study. A *post hoc* analysis with exclusion of patients with major protocol violations, dual RAS blockade or haematuria increased the ACR difference between the two treatment arms to 32% at Week 12 (P = 0.014) and 39% at Week 20 (P = 0.010).

**Conclusions:** Inhibition of the CCL2/CCL2 receptor axis with emapticap pegol was generally safe and well tolerated. Beneficial effects on ACR and HbA1c were observed in this exploratory study, which were maintained after cessation of treatment. Taken together, emapticap may have disease-modifying effects that warrant further investigation in adequately powered confirmatory studies.

## INTRODUCTION

Type 2 diabetes remains the leading cause (>40%) of new patients requiring dialysis [1]. Diabetic nephropathy (DN) develops over many years and is characterized by the gradual increase in albuminuria and decline in renal function. The renal and the cardiovascular risk of DN patients can be reduced with angiotensin-converting enzyme inhibitors (ACEis) or angiotensin receptor blockers (ARBs), and the protective effect of these drugs has at least partly been attributed to their albuminuria-lowering effect [2]. However, there is a great need for novel treatment modalities because the residual renal and cardiovascular risk of this patient population remains high. It has been demonstrated that interstitial macrophage infiltrates are common in DN [3] and in patients and animals, the pro-inflammatory chemokine C-C motif-ligand 2 (CCL2), also called monocyte-chemotactic protein 1 (MCP-1), is implicated in the development of insulin resistance [4], as well as macrophage infiltration [3]. The role of macrophages in inflammation and even proteinuria has been underscored in diabetic mouse models [5, 6]. More recently, it was shown that urinary CCL2 levels are elevated in women before clinical findings of DN, underlying the potential importance of inflammatory processes in the pathophysiology of the disease [7, 8]. A CCL2 antagonizing l-RNA aptamer (Spiegelmer) reduced glomerular macrophages by 40%, improved diffuse glomerulosclerosis and inhibited decline in glomerular filtration rate in uninephrectomized db/db mice [9]. Blocking the CCL2 receptor CCR2 with other compounds produced similar results in the db/db model [10–13]. These data have substantiated the hypothesis that blockade of the CCL2/CCR2 axis might be a meaningful new therapeutic target to treat patients with diabetic kidney injury [14, 15]. Emapticap pegol (NOX-E36) is a 40-nucleotide oligonucleotide aptamer that binds and inhibits CCL2 with high affinity and specificity. Emapticap neither hybridizes with native nucleic acids nor activates the innate immune response and was well tolerated in Phase I human trials [16, 17]. We report the results of an exploratory Phase IIa study in diabetic patients with albuminuria.

## MATERIALS AND METHODS

**FIGURE 1:**
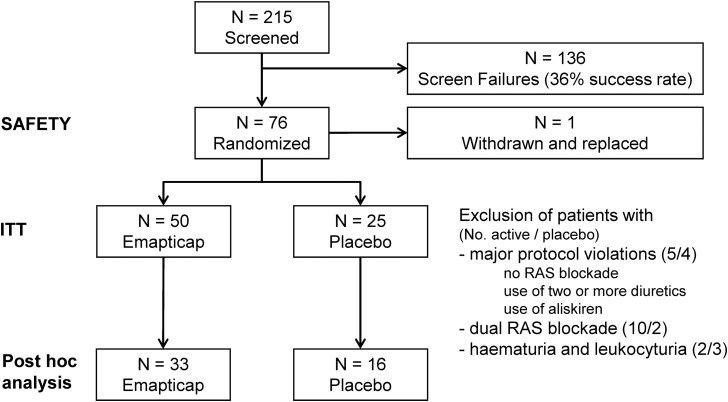
Consolidated Standards of Reporting Trials (CONSORT) diagram: summary of the disposition of study participants in the ITT population and a *post hoc* analysis excluding patients with major protocol violations, dual RAS blockade or haematuria and leukocyturia.

This exploratory trial was conducted as a double-blind, randomized multi-centre study in five European countries. Seventy-six patients with DN were included with a 2:1 randomization to emapticap or placebo (Figure 1). The primary objective was to characterize the effect of emapticap on the change in urinary albumin/creatinine ratio (ACR). Secondary objectives included evaluation of the effect on glycaemic control as well as safety and tolerability. The study protocol was in accordance with the Declaration of Helsinki (2002) and was approved by local and central review boards (ClinicalTrials.gov Identifier: NCT01547897). The main eligibility criterion was type 2 diabetes according to American Diabetes Association definition with an HbA1c from 6.0 to 10.5%. Patients had received stable anti-diabetic, anti-hypertensive and lipid-lowering medication for 3 months prior to screening, including therapy with an ACEi and/or an ARB. They had an ACR >100 mg/g in at least two out of three morning void urines and an estimated glomerular filtration rate (eGFR) >25 mL/min/1.73 m^2^ according to the Chronic Kidney Disease Epidemiology Collaboration (CKD-EPI) formula. The major exclusion criteria were uncontrolled hypertension (>180/110 mmHg), a cardiovascular event or acute kidney injury in the last 3 months as well as treatment with aliskiren, two or more diuretic drugs, systemic non-steroidal anti-inflammatory drugs or thiazolidinediones.

**FIGURE 2:**
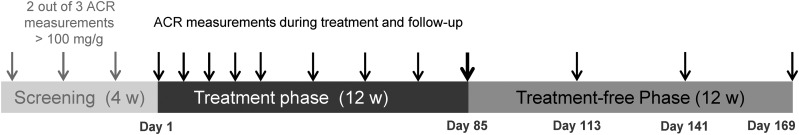
Study design.

Emapticap was administered subcutaneously at 0.5 mg/kg twice weekly for 85 days, followed by a treatment-free observation period of 12 weeks (Figure 2). During the treatment phase, ACR was determined as single measurements weekly or every other week and during the follow-up every 4 weeks until Day 169, i.e. 12 weeks after treatment cessation. Urine samples for ACR determination were shipped at ambient temperature to the central lab and were analysed upon arrival using an immunoturbidimetric assay (Roche Modular). HbA1c was measured every 4 weeks until Day 113, i.e. 4 weeks after treatment cessation. The complete clinical chemistry was measured in a central laboratory (BARC, Belgium). Emapticap levels were measured by NOXXON Pharma AG, Berlin, Germany.

**FIGURE 3:**
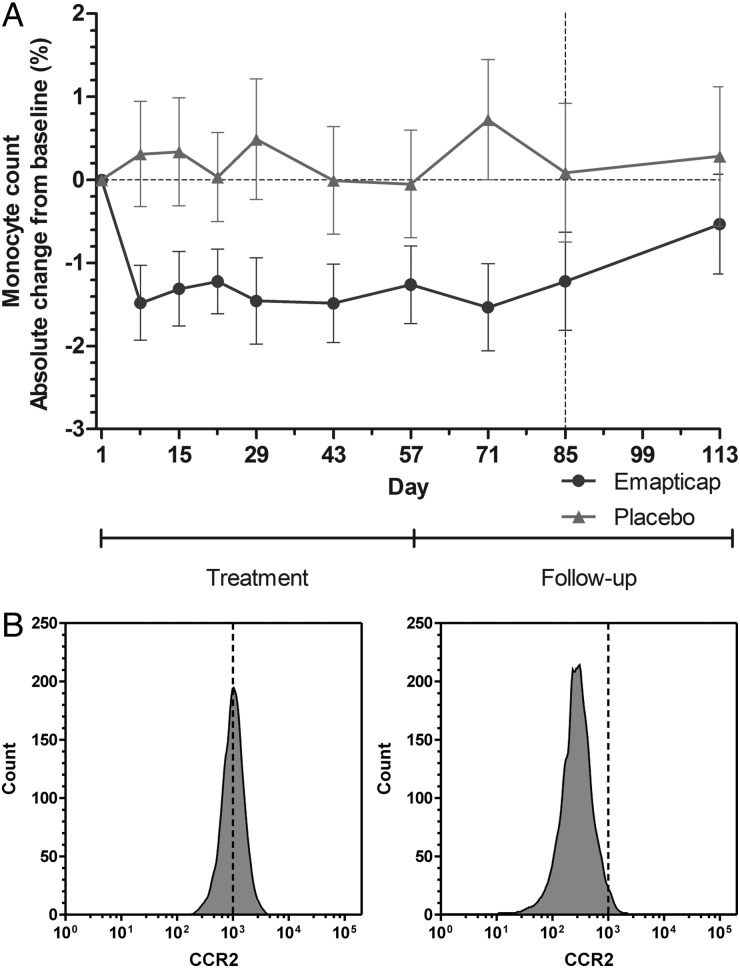
(**A**) Time course of monocyte count change from baseline (ANCOVA; ari-LS-means with 95% CI). (**B**) Flow cytometry of exemplary patient pre-dose (Day 1) and after 4 weeks of dosing (Day 29).

### Statistical analysis

This study was designed as an exploratory proof of concept study and no formal sample size calculation was performed prior to the start of the study. The primary objective was to characterize the effect of study drug on the change in ACR (Week 12 minus baseline) and this was compared with placebo by analysis of covariance (ANCOVA) using baseline values as covariate. As the parameter ACR is not normally distributed, a logarithmic transformation was used to calculate mean changes from baseline and respective 95% confidence intervals (CI). These were back-transformed to provide geometric means with respective 95% CI. Descriptive statistics are provided for all continuous study variables and categorical data are described by absolute and relative frequencies. All statistical analyses were performed using SAS^®^ software version 9.1.3 or later (SAS Institute, Cary, NC, USA). Independent statisticians performed the interim analyses. During a treatment period of 4 weeks, interim data from 12 patients were reviewed in a blinded fashion in order to confirm the predicted pharmacokinetic/pharmacodynamic profile (e.g. flow cytometry of monocytes) of the study drug. After completion of the treatment phase of 27 and 51 patients, a pre-specified interim analysis for the efficacy parameters and eGFR was performed.

Safety was assessed for all 76 patients who received at least one dose of study medication. For the intent-to-treat (ITT) analysis, the data from all 75 patients for whom both baseline data and data on the primary efficacy variable for at least one post baseline visit were available were analysed. For the assessment of albuminuria, we also performed a *post hoc* analysis. For this analysis, we excluded patients with major protocol violations, treatment with dual RAS blockade or haematuria and leukocyturia (Figure 1) from the ITT population.

Statistical analyses were performed by the clinical research organization (CRO) with validation by a biostatistician-advisor of the sponsor. The authors had complete control over the analysis.

## RESULTS

### Pharmacokinetics and pharmacodynamics

Recruitment began in March 2012, the first patient entered the study in June 2012 and the study was completed in December 2013. The baseline characteristics for the ITT population are summarized in Table 1. The treatment duration was 12 weeks. After 2 weeks of treatment, steady-state plasma concentrations within the therapeutic range of 355 ± 105 nM were reached (Supplementary data, Figure S1). The blood monocyte count was reduced by 15–20% within 1 week after treatment was commenced and remained lower than in the control group throughout the study period (Figure 3A). Four weeks after stopping treatment, this difference was markedly reduced; in addition, the density of the CCL2 receptor CCR2 on the surface of the monocytes was 4- to 5-fold reduced (Figure 3B).

**Table 1:** Patient characteristics at baseline for the ITT population

	Emapticap (*N* = 50)	Placebo (*N*= 25)	P-value *t*-test^d^
Male gender, *n* (%)	39 (78)	18 (72)	0.578^e^
Age (years)^a^	61.5 (5.7)	61.0 (8.4)	0.761
Body weight (kg)^a^	94.9 (17.8)	105.8 (26.4)	0.038
BMI (kg/m^2^)^a^	33.2 (6.1)	35.9 (6.7)	0.090
Duration of diabetes (years)^a^	12.4 (5.7)	14.5 (7.5)	0.177
Fasting plasma glucose (mg/dL)^b^	165 (152.5–178.8)	211 (186.0–238.3)	0.001
HbA1c (%)^b^	7.9 (7.58–8.19)	8.1 (7.68–8.53)	0.404
Supine blood pressure (mmHg)^b^			
Systolic	142 (138.2–146.4)	142 (136.9–148.1)	0.963
Diastolic	79 (77.0–80.9)	78 (74.8–80.2)	0.373
Urinary ACR (mg/g)^b^	589 (428–809)	968 (551–1701)	0.096
Urinary ACR (mg/g)^c^	531 (244–1432)	834 (311–2385)	0.096
Serum creatinine (µmol/L)^b^	101.0 (94.2–108.5)	96.8 (84.2–111.2)	0.540
eGFR CKD-EPI (mL/min/1.73 m^2^)^b^	64 (58.2–70.7)	65 (55.1–77.3)	0.844
RAS blockade, *n* (%)	49 (98)	25 (100)	–
ACEi only	21 (42)	17 (68)	
ARB only	18 (36)	6 (24)	
ACEi + ARB	10 (20)	2 (8)	

^a^Arithmetic mean (SD).

^b^Geometric mean (95% CI).

^c^Median (Q1 to Q3).

^d^Based on log values for geometric means.

^e^Fisher's exact test.

### Albuminuria

**FIGURE 4:**
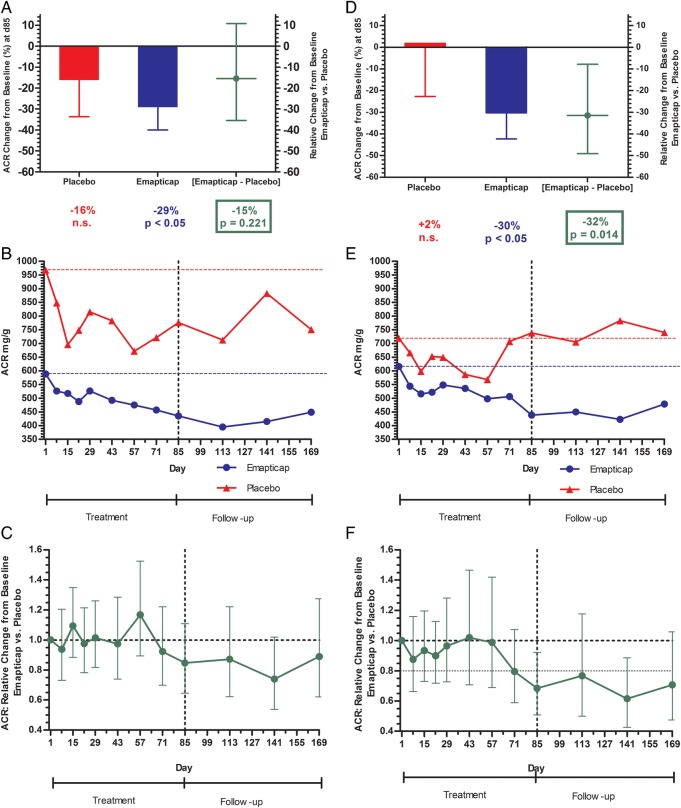
Change in urinary ACR in the ITT population (**A**–**C**) and the *post hoc* analysis set (**D**–**F**). (A and D) Relative ACR change from baseline versus placebo at end of treatment (ANCOVA; geo-LS-means and geo-LS-mean ratio with 95% CI); (B and E) absolute ACR time course for emapticap and placebo during and after treatment; (C and F) relative change from baseline versus placebo (ANCOVA; geo-LS-mean ratio with 95% CI).

At baseline, we observed a non-significant imbalance in the urinary ACR between the two treatment groups (Table 1). Treatment with emapticap lowered the ACR significantly by 29% (P < 0.05) at Day 85 versus baseline (Figure 4A). In the placebo group, the ACR decreased non-significantly by 16%. Compared with placebo, there was a trend towards reduction of ACR by 15% (95% CI: 10.8 to −35.5%; P = 0.221) in an ANCOVA analysis. The time course of ACR during and after treatment with emapticap is illustrated in Figure 4B. During the first 2 months of treatment, both placebo and emapticap groups showed a parallel decrease from baseline, whereas a separation became apparent after Day 57. The therapeutic effect of emapticap was maintained after the cessation of dosing until the end of the 3-month treatment-free observation period (Figure 4B and C). The maximum effect on mean ACR, a 40% reduction versus baseline (P < 0.001) and a 26% reduction versus placebo (P = 0.064), were observed 4 and 8 weeks after the last dose, respectively. RAS blockade and diuretic treatment remained stable during the study period.

Double RAS blockade and nephritic syndrome, i.e. the presence of haematuria and leukocyturia in addition to albuminuria, are known to be confounding factors of ACR. Therefore, 12 patients who had received an ACEi and an ARB, and 5 patients who had presented with nephritic syndrome at baseline were excluded for a *post hoc* analysis, in addition to those with major protocol violations. In fact, the baseline difference in ACR between the placebo and emapticap group was markedly reduced from 1.64-fold in the ITT population to 1.17-fold in this *post hoc* analysis set of 49 patients (Supplementary data, Table S1). In this subgroup of patients, emapticap treatment reduced ACR by 30% (P < 0.05) versus baseline and 32% (P = 0.011) versus placebo at Day 85 (Figure 4D). An ACR reduction of ≥50% was observed in 31% versus 6% (P = 0.058) (Supplementary data, Figure S2). Eight weeks after treatment had been stopped, we observed a relative ACR reduction of 39% (95% CI: −11.4 to 57.3%; P = 0.010) in comparison with the placebo arm (Figure 4E and F).

When comparing the ITT and *post hoc* analysis data, it is obvious that (i) qualitatively, both sets show a virtually identical time course of ACR—the specific beneficial effect sets in 2 months after start of treatment, improves further until end of treatment and is maintained after cessation of dosing; (ii) quantitatively, the placebo-corrected effect of emapticap on ACR is most pronounced and statistically significant in the *post hoc* analysis, and maintained at least as a clear trend in the ITT population; and (iii) the improved separation in ACR between emapticap and placebo in the *post hoc* analysis is mainly due to reduced background noise in the placebo group.

**FIGURE 5:**
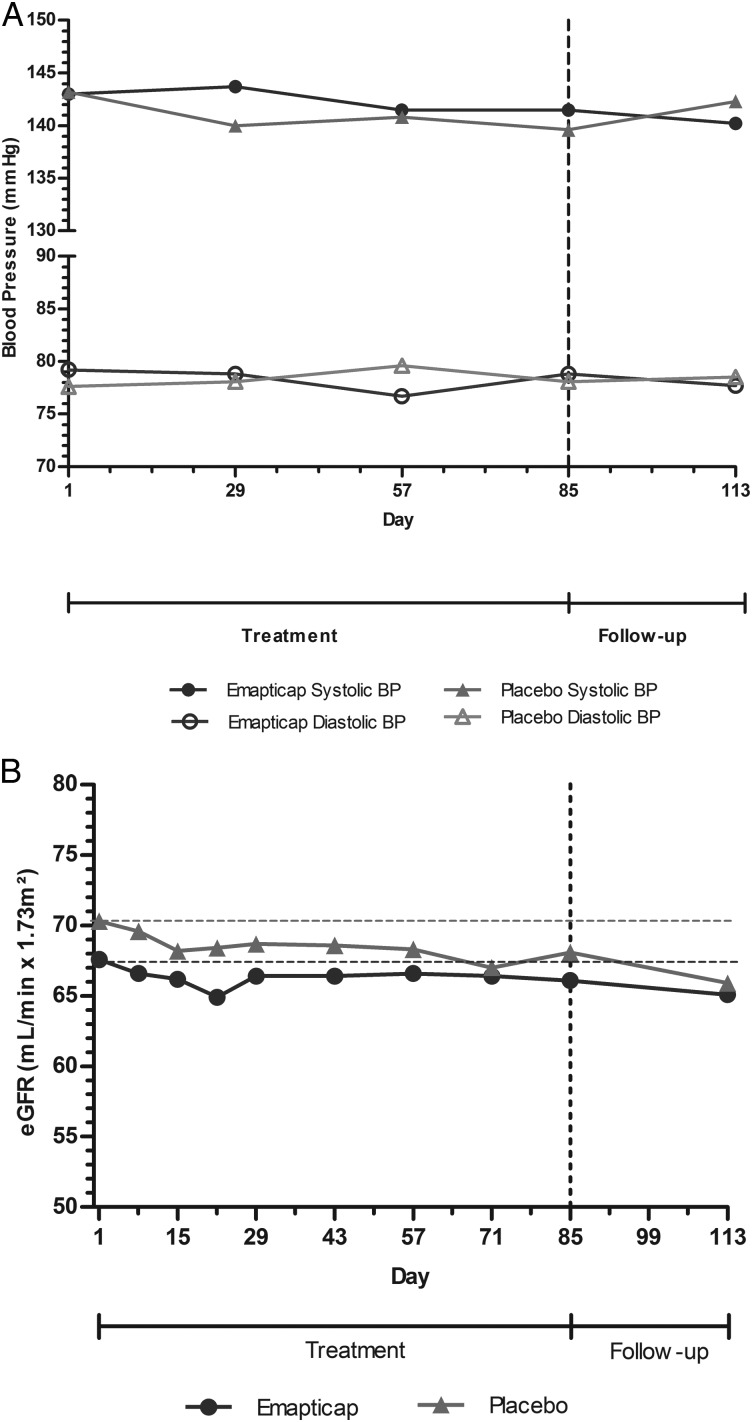
Change in systolic and diastolic blood pressure (**A**) and eGFR (**B**) in the ITT population.

No relevant difference in systolic or diastolic blood pressure (Figure 5A) was seen between the treatment groups throughout the study. In the emapticap group, the mean systolic and diastolic blood pressure had changed by −1.5 and −0.4 mmHg at Day 85 and in the placebo group by −3.6 and +0.5 mmHg, respectively. We observed a small comparable eGFR drop in both groups of −1.5 and −2.2 mL/min at Day 85 for emapticap and placebo, respectively (Figure 5B).

### HbA1c

**FIGURE 6:**
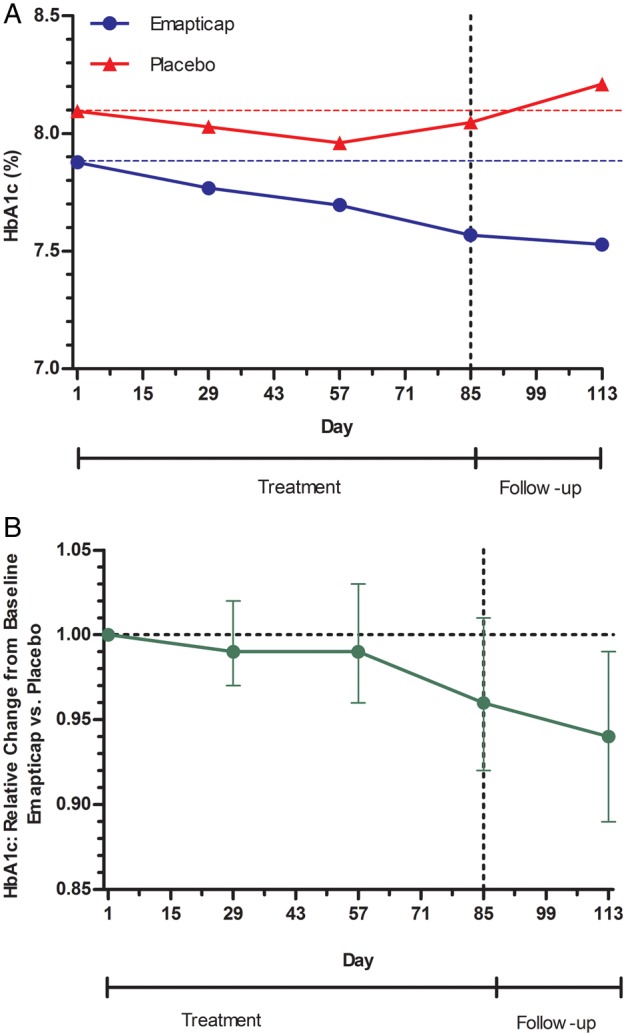
Change in HbA1c in the ITT population. (**A**) Absolute HbA1c time course for emapticap and placebo during and after treatment. (**B**) Relative HbA1c change from baseline versus placebo (ANCOVA, geo-LS-mean ratio with 95% CI).

HbA1c levels were significantly reduced versus baseline levels by 0.31% (P = 0.014) in the emapticap group and non-significantly in the placebo group (0.05%; P = 0.843; Figure 6A). The difference between the two groups showed a trend towards improvement (P = 0.146). The time course of absolute change in HbA1c during and after dosing is illustrated in Figure 6A. Emapticap showed a sustained steady decrease from Day 1 to Day 85, whereas placebo revealed a slight initial decrease to Day 57 with subsequent re-increase to baseline. Four weeks after the last dose (Figure 6), we found a significant difference between the two treatment arms (−0.35 versus +0.12%; P = 0.026). The concomitant change in HbA1c (on a log scale) had no significant effect on the ACR change from baseline (P = 0.3142).

### Safety

Emapticap pegol was generally safe and well tolerated. No treatment-related serious adverse event (AE) occurred during the treatment and the follow-up phase. Three patients stopped treatment prematurely because of AEs: two with treatment-related skin reactions and one patient with urinary ACR increase thought to be unrelated to treatment. In Table 2, all AEs are listed. Most AEs were mild. The only relevant treatment-related AEs were generally mild local injection site reactions, which occurred in 18 and 4% of patients treated with emapticap or placebo, respectively. The number of AEs of severe intensity was balanced between emapticap and placebo, and was of the type commonly observed in this population. Cardiac disorders [unstable angina (*n* = 1), atrioventricular block first degree (*n* = 1) and ventricular extrasystoles (*n* = 1)] and nervous system disorders [dizziness (*n* = 2), disturbance in attention (*n* = 1) and headache (*n* = 1)] were observed sporadically and only in patients treated with emapticap. Taking into account the small sample size and the fact that twice as many patients were treated with emapticap, this asymmetric distribution is considered to be incidental, typical for the underlying disease and not treatment related. The abnormal clinical laboratory tests (increases and decreases in haematology, coagulation and clinical chemistry tests) reflected common findings in the type 2 diabetic population without a specific direction and relevant differences between treatment groups. ECG, vital signs and physical examination showed changes also typical for the population under study. No relevant difference in systolic or diastolic blood pressure was seen between the treatment groups throughout the study. At Day 85, the mean systolic/diastolic blood pressure had changed by −1.8/0.2 mmHg and by −2.2/1.1 mmHg for emapticap and placebo, respectively. No relevant change of body weight occurred during the study.

**Table 2:** Summary of AEs

Adverse event	Emapticap (*N* = 51), %	Placebo (*N* = 25), %	Total (*N* = 76), %
Any adverse event	51	36	46
Any serious adverse event (e.g. humerus fracture, erysipela, unstable angina, hypertension, diabetic foot, appendicitis)	10	8	9
Infections	18	20	18
General disorders and administration site disorders (e.g. hematoma, pain, erythema, oedema)	18	8	15
Musculoskeletal and connective tissue disorders	10	12	11
Gastrointestinal disorders	8	8	8
Injury, poisoning and procedural complications	6	12	8
Metabolism and nutrition disorders	8	4	7
Investigations (clinical laboratory tests)	6	4	5
Vascular disorders	6	4	5
Cardiac disorders	6	0	4
Nervous system disorders	6	0	4
Skin and subcutaneous tissue disorders	4	4	4
Respiratory, thoracic and mediastinal disorders	2	4	3
Blood and lymphatic system disorders	2	0	1
Eye disorders	0	4	1

## DISCUSSION

Twelve weeks of treatment with 0.5 mg/kg emapticap was generally safe and well tolerated in patients with DN. There was a trend towards reduced urinary albumin excretion and HbA1c. Four features of this treatment response were particularly intriguing: (i) that more than 2 months of treatment was required before the beneficial effects became detectable versus placebo; (ii) the maintenance of the effect on ACR after treatment cessation; (iii) the independence of the effect on ACR from relevant blood pressure or eGFR changes; and (iv) the combination of potential renoprotective and anti-diabetic activity. Such a profile is clearly different from other agents known to decrease albuminuria such as RAS blockers or endothelin receptor antagonists, which are characterized by a more rapid effect on proteinuria, blood pressure lowering and an abrupt return to baseline after discontinuation [18–20]. We believe that the common denominator for emapticap's response profile is an anti-inflammatory mode of action. Our data suggest that emapticap reduces the amount of circulating monocytes. This is in agreement with a previous animal study which showed that a CCL2 antagonizing Spiegelmer reduces the peripheral monocyte count by inhibiting the CCR2-positive monocyte emigration from the bone marrow to the blood [21]. Additionally, we observed a reduction of the CCR2 density on the surface of the monocytes. These two alterations should disable the monocytes from accumulating in organ tissue and might explain the effects observed in our study.

Recently, the role of monocyte/macrophage infiltration in the pathogenesis of diabetic organ complications has been of increasing interest [3]. Several animal studies and indirect evidence from human studies have suggested that the inflammation associated with macrophage influx is critical for the development of progressive kidney injury [3]. Furthermore, mouse studies have indicated that the CCL2/CCR2 signalling cascade is critical and blockade of this axis by different approaches was able to improve proteinuria as well as progression of renal damage [9–13]. Monocytes contribute to glomerular damage and may induce matrix deposition and induction of fibrosis. The present study suggests that these very promising results in animal models translate well into human DN. At end of 3 months of treatment, we observed a 29% reduction of albuminuria from baseline and 15% versus placebo, whereas the maximum effect of 40% versus baseline and 26% versus placebo was found 4–8 weeks after cessation of treatment. Taking the short treatment duration into account this result compares favourably with other approaches like aliskiren and paricalcitol, which showed 20 and 18% reduction on top of RAS blockade after 6 months of treatment, respectively. The currently most progressed and efficacious approach, the endothelin antagonist atrasentan, showed a comparable effect size on urinary ACR from baseline (36.2% reduction for the 0.75 mg/day dose) after 3 months of treatment [19]. However, in contrast to most of the drugs mentioned and all RAS inhibitors, emapticap's effect on urinary albumin excretion is not associated with changes of blood pressure or eGFR. This is in line with the findings in a study with CCX140-B that targets the CCL2 receptor where the reported reduction of urinary ACR (16% versus placebo for the 5 mg dose and 10% for the 10 mg dose) was independent of haemodynamic changes [22]. Administration of the other drug classes led to a fast reduction of albuminuria within 2–4 weeks [18–20, 23], a blood pressure drop [18–20, 23, 24] and a decline of the eGFR [18–20, 24], suggesting that all of these drugs have a significant effect on systemic and/or intrarenal haemodynamics. The absence of any relevant changes of blood pressure/eGFR and the maintenance of the effect on ACR for several weeks after cessation of treatment are in line with emapticap's anti-inflammatory mode of action. These data suggest that CCL2 blockade influences important functional or structural pathophysiological mechanisms of DN, which differentiates CCL2 blockade from the existing therapeutic strategies and indicates the disease-modifying potential of this approach. A meta-analysis of 21 trials with the aim to delineate the association between changes in albuminuria and end-stage renal disease found an association of interventions that reduced albuminuria by at least 15% during the first months of treatment and improved hard renal outcomes [25]. The observed magnitude of the ACR reduction on top of standard of care therefore suggests that emapticap has renoprotective potential.

Recent studies in mice have suggested uniformly that blockade of the CCL2 receptor CCR2 will lead to an improvement of insulin resistance and glycaemic control [11–13]. The data obtained in the current study suggest that blockade of CCL2 is indeed able to improve hyperglycaemia in patients with type 2 diabetes. The notable reduction of absolute HbA1c levels after 3 months of treatment by 0.3–0.4% on top of standard diabetes drugs may be attributed to changes in the inflammatory milieu of the pancreatic islet cells and/or the adipose tissue. We cannot exclude that the positive effect on HbA1c might have contributed partially to the effect on albuminuria. In the ACCORD study, an HbA1c reduction of 1.3% in the intensive treatment arm was accompanied by 12.5% lower urinary ACR levels in comparison to the standard glycaemia control arm [26]. However, Levin *et al.* [27] observed no effect on urinary ACR levels after 1-year intensive glycaemic control in type 2 diabetic patients with microalbuminuria. We did not observe a significant effect of the HbA1c changes on the degree of albuminuria. In contrast, the anti-inflammatory drug salicylate (salsalate) also reduced HbA1c in type 2 diabetic patients by a similar degree of 0.37%, but increased albuminuria [28], suggesting that the mode of action on the inflammatory milieu is different between the two treatment strategies.

We are aware that this exploratory study has limitations. First, the influence of confounding factors on the volatile parameter urinary ACR can be expected to be substantial. One of these confounding factors is dual RAS blockade, which had been received by 16% of the patients in the study. Dual RAS blockade reduces albuminuria to a greater extent than single RAS blockade [29], but has been linked to worse renal outcome [30] even in macroalbuminuric patients [31, 32]. As a consequence, dual blockade has been removed from the 2013 published European Society of Hypertension guidelines and is now even labelled as contraindicated [33]. Another confounding factor of the ACR is nephritic syndrome, i.e. the triad of albuminuria, haematuria and leukocyturia, which indicate the presence of an additional, more acute inflammatory status [34, 35]. In an attempt to exclude these confounders, we performed a *post hoc* analysis of the per-protocol set without patients on dual RAS blockade and patients with nephritic syndrome, and in fact the change in ACR became significant versus placebo in this patient subset. It is important to note that only the ACR time course in the placebo group was markedly different in this subset compared with the ITT population, whereas there was only minor change in the emapticap group. This signal of a potential renoprotective effect of emapticap remains to be verified in an adequately powered study with concrete endpoints. Second, although this was a randomized study, several baseline characteristics were not balanced between the groups due to the small sample size. This imbalance may have contributed to a worse renal and metabolic outcome in the placebo group, which had significantly higher plasma glucose level and non-significantly higher urinary ACR and HbA1c at baseline as well as higher body mass index and longer duration of diabetes.

Our results suggest an important role of CCL2 and inflammatory mechanisms in the pathogenesis of DN. Emapticap is a novel approach with potential for treating DN. As the need is great and the therapeutic options are limited, we suggest that the utility of emapticap should be assessed in larger trials.

## SUPPLEMENTARY DATA

Supplementary data are available online at http://ndt.oxfordjournals.org.

## Supplementary Material

Supplementary Data
