# Supplementary material for: C-C motif-ligand 2 inhibition with emapticap pegol (NOX-E36) in type 2 diabetic patients with albuminuria
Source: Nephrol Dial Transplant. 2016 Apr 27;32(2):307–15. doi: 10.1093/ndt/gfv459 (PMC5410979; doi:10.1093/ndt/gfv459)
Supplement: Supplementary Data [file gfv459_Supp.docx]

**Supplementary Appendix**

**CCL2 Inhibition with Emapticap Pegol (NOX-E36) in Type 2 Diabetic Patients with Albuminuria**

**Table S1. Baseline characteristics post hoc set analysis set**

|  | **Post Hoc**  **Analysis Set** | | |
| --- | --- | --- | --- |
|  | **Emapticap**  **(N=33)** | **Placebo**  **(N=16)** | **p-value**  **t-Test^§^** |
| Male gender, n (%) | 25 (76) | 14 (87) | 0.464^$^ |
| Age [yrs]* | 61.7 (5.9) | 60.1 (9.0) | 0.460 |
| Body weight [kg]* | 91.8 (15.9) | 112.4 (28.4) | 0.002 |
| Body Mass Index [kg/m^2^]* | 31.8 (5.4) | 37.0 (7.1) | 0.007 |
| Duration of diabetes [yrs]* | 12.1 (5.9) | 14.8 (8.4) | 0.206 |
| Fasting plasma glucose [mg/dL]** | 163  (145.1 to 181.9) | 198  (169.7 to 231.4) | 0.041 |
| HbA1c [%]** | 7.8  (7.49 to 8.21) | 7.9  (7.45 to 8.45) | 0.760 |
| Supine blood pressure [mmHg]**  Systolic  Diastolic | 144  (139.1 to 148.7)  80  (77.5 to 82.2) | 143  (135.9 to 149.6)  79  (75.8 to 82.7) | 0.770  0.746 |
| Urinary ACR [mg/g]** | 616  (399 to 952) | 719  (377 to 1373) | 0.681 |
| Urinary ACR [mg/g]*** | 535  (218 to 1701) | 694  (290 to 1658) | 0.681 |
| Serum creatinine [µmol/L]** | 98.8  (90.9 to 107.3) | 93.2  (77.6 to 112.0) | 0.493 |
| eGFR CKD-EPI [mL/min]** | 66  (58.7 to 73.4) | 71  (58.1 to 86.5) | 0.454 |
| RAS blockade, n(%)  ACEi only  ARB only | 33 (100)  18 (55)  15 (45) | 16 (100)  10 (63)  6 (38) | - |

*Arithmetic mean (SD), **Geometric mean (95% CI), ***Median (Q1 to Q3), § Based on log values for geometric means, $ Fisher exact test

# Figure S1. Pharmacokinetics of emapticap pegol


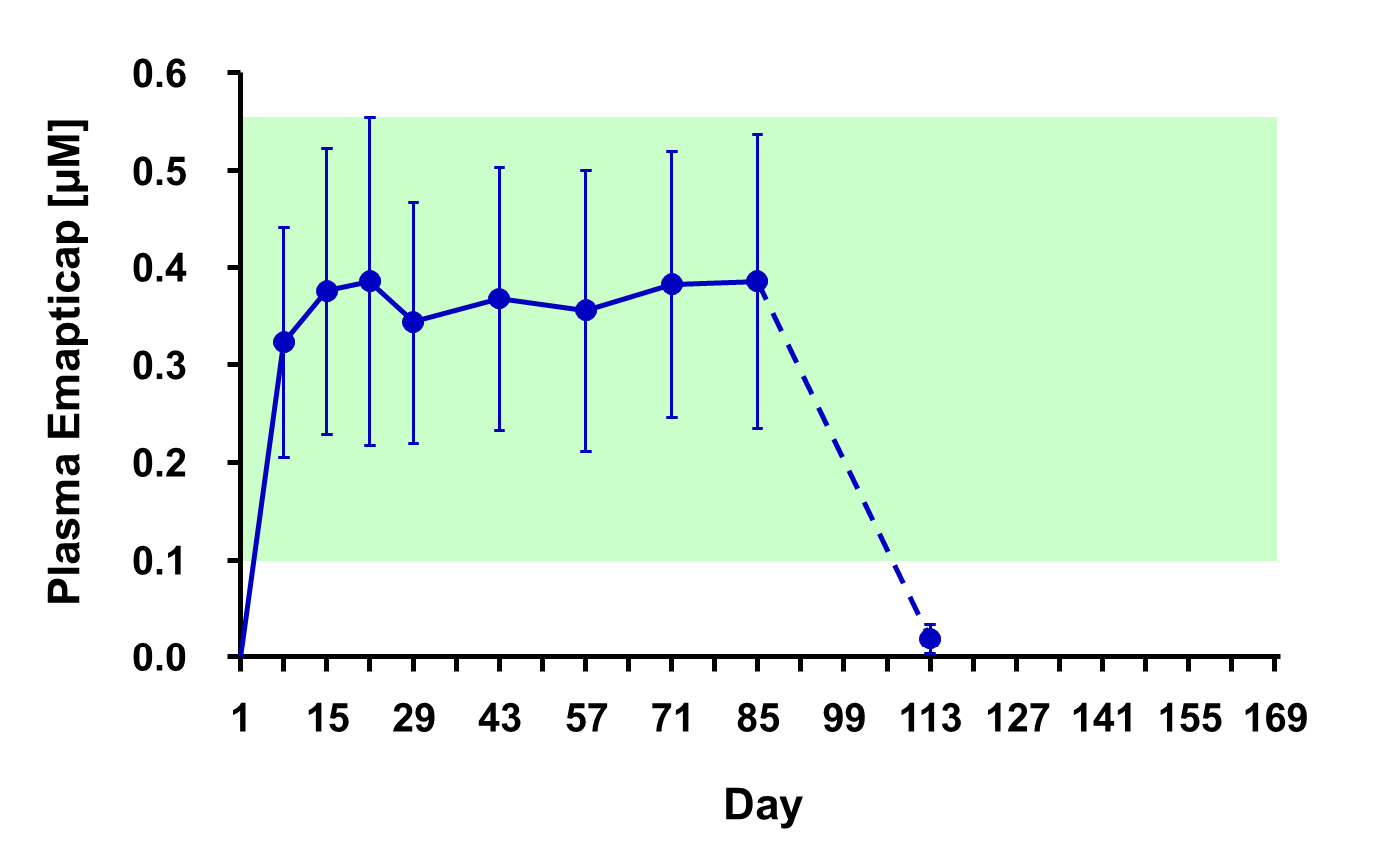


**Figure S2. Individual ACR response** at end of treatment (day 85) in the post hoc analysis set

**A**

**
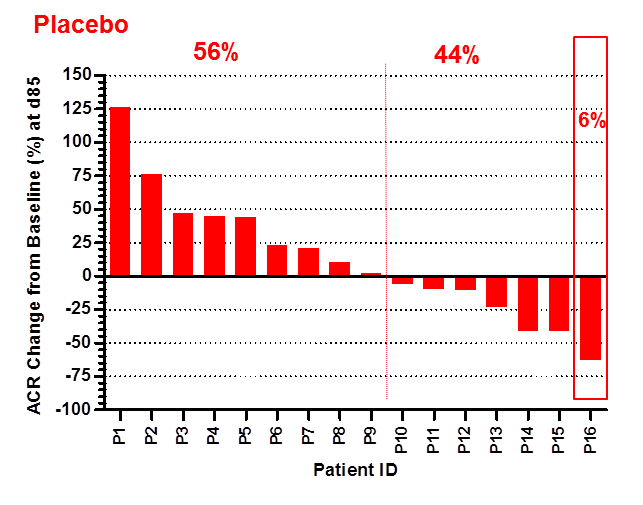
**

**B**

**
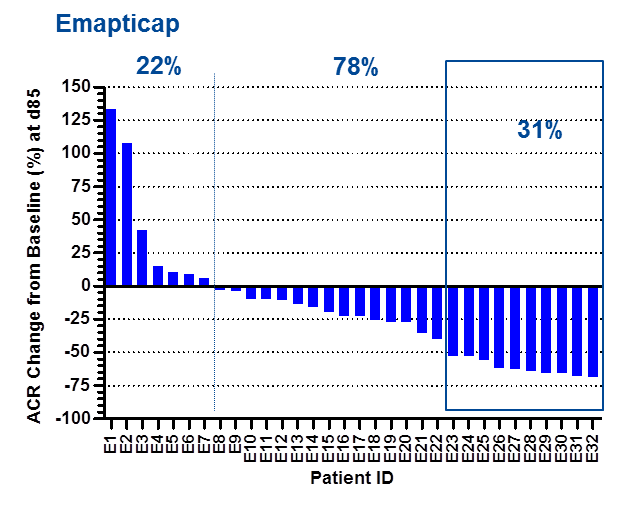
**
